# Identification of Concomitant Inhibitors against Glutamine Synthetase and Isocitrate Lyase in *Mycobacterium tuberculosis* from Natural Sources

**DOI:** 10.1155/2022/4661491

**Published:** 2022-10-03

**Authors:** Anesha Chanda, Sanjib Kalita, Awdhesh Kumar Mishra, Liza Changkakoti, Janayita Biswa Sarma, Kunal Biswas, Debashree Kakati, Yugal Kishore Mohanta, Bhaben Tanti, Saurov Mahanta, Muthupandian Saravanan

**Affiliations:** ^1^National Institute of Electronics and Information Technology, Guwahati, 781008 Assam, India; ^2^Department of Botany, Gauhati University, Jalukbari, Guwahati, 781014 Assam, India; ^3^Department of Biotechnology, Yeungnam University, Gyeongsan 38541, Republic of Korea; ^4^Assam Science and Technology University, Jalukbari, Guwahati, 781014 Assam, India; ^5^Centre for Nanoscience & Nanotechnology International Research Centre, Sathyabama Institute of Science and Technology, Jeppiaar Nagar, Rajiv Gandhi Salai, Chennai 600119, India; ^6^Department of Botany, Mangaldai College, Mangaldai, 784125 Assam, India; ^7^Department of Applied Biology, School of Biological Sciences, University of Science and Technology Meghalaya, 9th Mile, Techno city, Baridua, Ri-Bhoi, 793101 Meghalaya, India; ^8^Department of Microbiology, Division of Biomedical Sciences, Mekelle University, Ethiopia; ^9^AMR and Nanotherapeutics Laboratory, Department of Pharmacology, Saveetha Dental College, Saveetha Institute of Medical and Technical Sciences (SIMATS), Chennai 600077, India

## Abstract

Tuberculosis (T.B.) is a disease that occurs due to infection by the bacterium, *Mycobacterium tuberculosis* (Mtb), which is responsible for millions of deaths every year. Due to the emergence of multidrug and extensive drug-resistant Mtb strains, there is an urgent need to develop more powerful drugs for inclusion in the current tuberculosis treatment regime. In this study, 1778 molecules from four medicinal plants, *Azadirachta indica*, *Camellia sinensis*, *Adhatoda vasica,* and *Ginkgo biloba,* were selected and docked against two chosen drug targets, namely, Glutamine Synthetase (G.S.) and Isocitrate Lyase (I.C.L.). Molecular Docking was performed using the Glide module of the Schrӧdinger suite to identify the best-performing ligands; the complexes formed by the best-performing ligands were further investigated for their binding stability via Molecular Dynamics Simulation of 100 ns. The present study suggests that Azadiradione from *Azadirachta indica* possesses the potential to inhibit Glutamine Synthetase and Isocitrate Lyase of *M. tuberculosis* concomitantly. The excellent docking score of the ligand and the stability of receptor-ligand complexes, coupled with the complete pharmacokinetic profile of Azadiradione, support the proposal of the small molecule, Azadiradione as a novel antitubercular agent. Further, wet lab analysis of Azadiradione may lead to the possible discovery of a novel antitubercular drug.

## 1. Introduction

Tuberculosis is an air-borne disease affecting the lungs, presenting symptoms like severe cough, chest pain, and fever. Tuberculosis (T.B.) is a contagious bacterial infection resulting in 48.3 cases per 100,000 inhabitants per year [1]. As per periodic reports published by World Health Organization (WHO), every year 2-3 million people lose their lives due to active Tuberculosis, while billion others carry dormant T.B. throughout the world. In 1882, German Microbiologist Robert Koch revealed that *M. tuberculosis* is the disease-causing agent of T.B. [2]. Over the decades, different antibiotics have been introduced to treat T.B.; however, the emergence of the drug-resistant strains of *M. tuberculosis* has imposed hurdles in the treatment and control of T.B. Multidrug-Resistant (M.D.R.) strains have resistance to potent T.B. drugs, including Rifampin and Isoniazid; Extensive Drug-Resistant (XDR) T.B. strains exhibit resistance against Isoniazid, Rifampin, Amikacin, Kanamycin, and Capreomycin, while Total Drug-Resistant (TDR) T.B. has resistance to all first-line and second-line T.B. drugs [3, 4]. Tuberculosis caused by MDR/XDR strains requires long-term treatment spanning over 9-20 months or even more, with a success rate below 50% globally [3]. The principal reason behind the requirement of long-term treatment in MDR/XDR TB is the smaller subpopulations of nonreplicating cells, which are not affected by standard antibiotics used and hence, require particular drugs to combat the deadly disease [5, 6].

In comparison to developed countries, T.B. is much more prevalent in developing countries. About 10.5 million people were infected with T.B. in 2015, and more than 1.4 million T.B. patients died, with 95% of deaths occurring in developing countries [7]. Human Immunodeficiency Virus (H.I.V.) coinfection with T.B. and the development of strains resistant to the most effective, 1^st^ line T.B. drugs are the two main factors responsible for the existing epidemic caused by T.B.

For the diagnosis and treatment of T.B., different strategies have been developed and are still being developed. In 1993, Directly Observed Treatment Short-Course (DOTS) was one of the most significant strategies among them. In 1998, DOTS plus was introduced to combat the multidrug-resistant (M.D.R.) strain [2, 7]. In the 1800s, “just sleep and eat nutritious food” was the advice given to T.B. patients [2]. In the beginning of the twentieth century, the *Bacillus* Calmette-Guerin (B.C.G.) vaccine was developed, saving many lives from this deadly disease. Until the discovery of Streptomycin in 1944, there was not even a single antitubercular drug [8]. But within a short period of time, Streptomycin also became ineffective in treating T.B., due to the emergence of Streptomycin-resistant strains. After that, different antitubercular drugs were introduced, including isoniazid (1952), pyrazinamide (1952), cysteine (1952), ethambutol (1962), rifampin (1957), and pyrazinamide (1957) [9]. However, due to the emergence of MDR-TB and XDR-TB strains, most commonly used antitubercular drugs became ineffective. Additionally, with the advancement of medical research in the last few years, scientists have obtained valuable insights into T.B. pathogenesis and treatment, yet further extensive research is required to decrease the incidence and subsequently eradicate T.B. successfully [2, 10]. Hence, there is an urgent need to identify new drug candidates in T.B. therapeutics [11].

According to the World Health Organization (WHO), “a medicinal plant” is any plant in which one or more of its organs contain substances that could be used for therapeutic purposes or as precursors for synthesizing suitable drugs. Plants are a repository of secondary metabolites which possess different medicinal properties. Plant secondary metabolites have caught much attention as drug candidates because of their less toxic nature [11]. Around 80% of the population in developing countries relies on traditional medicines derived from plants for their primary health care. In Ayurveda, different diseases, including Tuberculosis are treated using various plants based on the bioactive compounds available [12].

Microorganisms are usually controlled by blocking specific essential enzymes, thereby inhibiting the various biosynthetic pathways in them. Glutamine Synthetase (G.S.) and Isocitrate Lyase (I.C.L.) are two essential enzymes for >the growth of *M. tuberculosis*, and are effective drug targets [13, 14]. G.S. enzyme (E.C. 6.3.1.2) of *M. tuberculosis*, catalyses the ATP-dependent reaction between ammonium and glutamate, resulting in glutamine, A.D.P., phosphate, and a proton. Four G.S. homologues are found in *M. tuberculosis*, of which only one, encoded by the gene *glnA1*, is expressed highly for the survival of *M. tuberculosis* in both *in vitro* and *in vivo* conditions [15–17]. This enzyme can alter the ammonia concentration in the host cells where the infection is prevalent; it affects the pH of the phagosome resulting in the prevention of the phagosome fusion with the lysosome [18, 19].

In the Glyoxylate shunt, Isocitrate Lyase (I.C.L.) catalyses the conversion of Isocitrate to Succinate and Glyoxylate. Malate Synthetase condenses Glyoxylate with acetyl-CoA to form malate [20]. Two T.C.A. cycle steps involving decarboxylation are reportedly by-passed by I.C.L. and Malate Synthase, which is considered an essential phenomenon for fungal and bacterial pathogenesis [21]. ICL1 and ICL2, two forms of the enzyme Isocitrate Lyase encoded by genes *icl11* and *icl12,* respectively, are also crucial for the growth, survival, and virulence of *M. tuberculosis* [14].

Based on their usage in traditional medicine and their documented antitubercular activity, four plant species, namely, *Azadirachta indica, Camellia sinensis, Adhatoda vasica,* and *Ginkgo biloba* were selected for the current study [21–25]. From the ligand database, that was prepared based on the selected medicinal plants, Computer-Aided Drug Discovery (CADD) protocols were used to identify compounds with inhibitory properties against the two targeted enzymes, Glutamine Synthetase and Isocitrate Lyase of *M. tuberculosis.*

## 2. Results and Discussions

### 2.1. Molecular Docking

The binding affinity of the ligands towards the two selected receptors, 2WGS and 1F61, was determined via the Molecular Docking study. The binding affinity was determined based on the docking score; the lower the docking score and the greater the affinity. It was observed that the bioactive compound Azadiradione showed the highest binding relationship towards 2WGS (binding affinity score -10.2) followed by 7-desacetyl-7-benzoylazadiradione, 7-desacetyl-7-benzoylgedunin, alpha-amyrin, and Gedunin with binding affinities, -10.1, -9.1, -8.8, and -8.7, respectively (Table 1). For the receptor 1F61, 7-desacetyl-7-benzoylazadiradione displayed the highest binding affinity (binding affinity score -9.8), followed by 7-desacetyl-7-benzoylazadiradione, Azadiradione, Gedunin, and alpha-amyrin with binding affinities, -9, -8.5, -8.2, and -8, respectively (Table 1). Based on the binding affinity and the ability to effectively inhibit more than one receptor, the ligand, Azadiradione, was selected for further analysis.

### 2.2. Interaction of Azadiradione with the Active Sites of 2WGS and 1F61

**Figure 1:**
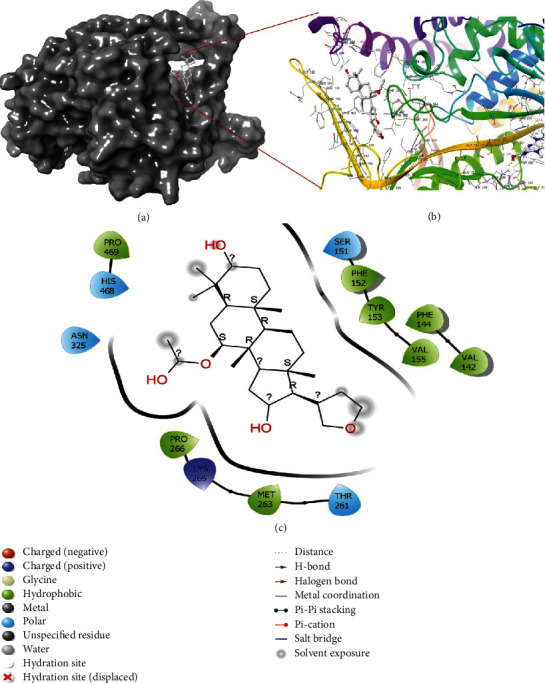
(a) Docking orientation of Azadiradione at the active site of 2WGS; (b) 3D-image of protein-ligand interaction generated using Maestro visualizer; (c) Docking pose and interaction of Azadiradione at the active site of 2WGS. The interaction diagram also illustrates the interactions at 0 ns of M.D. Simulation.

**Figure 2:**
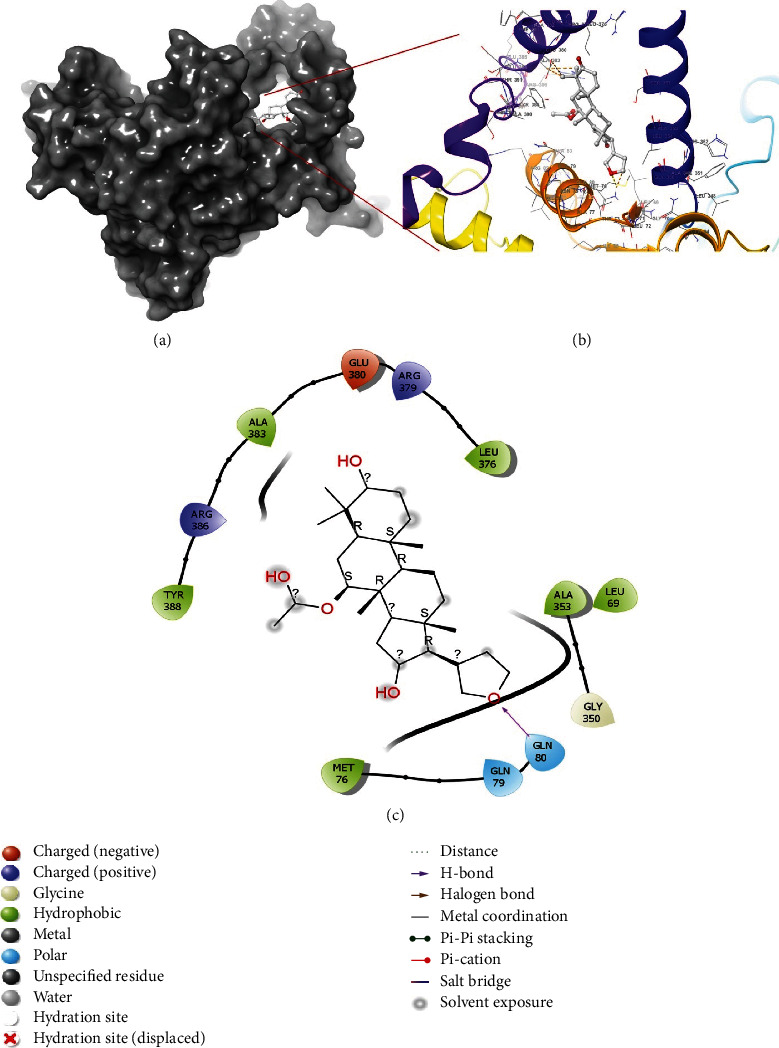
(a) Docking orientation of Azadiradione at the active site of 1F61; (b) 3D-image of protein-ligand interaction generated using Maestro visualizer; (c) Docking pose and interaction of Azadiradione at the active site of 1F61. The interaction diagram also illustrates the interactions at 0 ns of M.D. Simulation.

The interaction of Azadiradione with the active sites of the two selected enzymes, 2WGS, and 1F61, was analyzed using Maestro Visualizer of Schrӧdinger Suite 2020-3. It was observed that no H-bonds were formed between Azadiradione and 2WGS (Figure 1(c)), while 3 H-bonds were formed between Azadiradione and the residue Gln 80 of 1F61, as shown in Figure 2(c).

### 2.3. Molecular Dynamics (M.D.) Simulation

Molecular Dynamics Simulations were applied to the protein-ligand complexes to test the stability of the interactions between Azadiradione and the binding sites in the proteins. A simulation of 100 ns was run to check the stability of the ligand-protein complexes. Throughout the 100-ns simulation period, the backbone Root-Mean-Square Deviations (R.M.S.Ds) were analysed to measure the structural and dynamic properties of the protein-ligand complexes.

**Figure 3:**
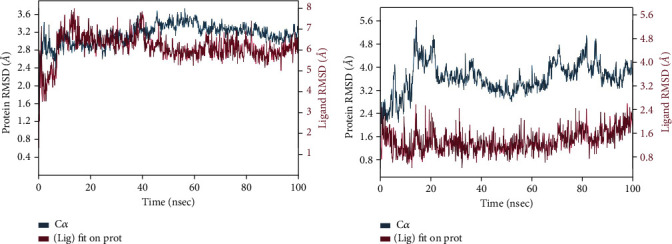
(a) R.M.S.D. plot of 2WGS and Azadiradione complex; (b) 1F61 and Azadiradione complex at 100 ns M.D. Simulation.

The R.M.S.D. plot for 2WGS and 2WGS bound to Azadiradione, presented in Figure 3(a), reveals the stability of the ligand-bound protein for the initial 10-40 ns of the M.D. Simulation; however, the structure displayed instability after 50 ns, followed by some stability around 100 ns. On the other hand, the R.M.S.D. plot for 1F61 and 1F61 bound to Azadiradione, presented in Figure 3(b), reveals the stability of both the protein and the ligand in the bound stage after 10 ns of M.D. Simulation.

**Figure 4:**
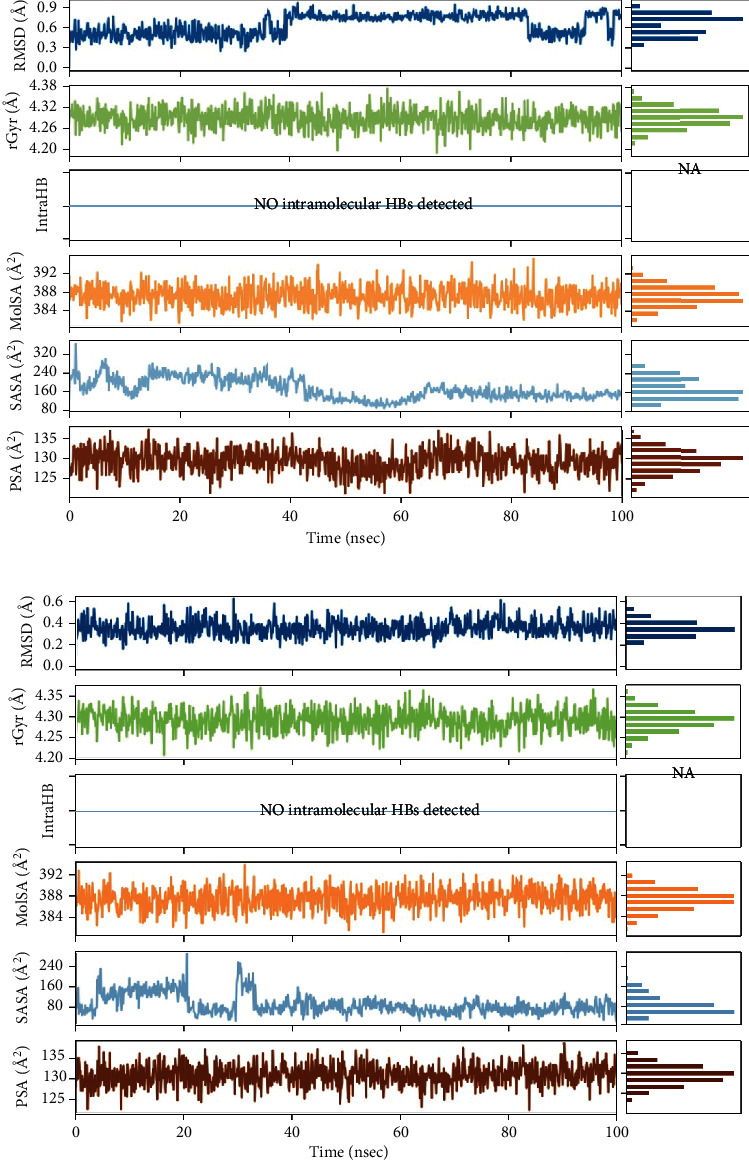
Ligand R.M.S.D.: Root-Mean-Square Deviation of ligands concerning the reference conformation. The radius of Gyration (rGyr): Representation of the “extendedness” of the ligands. Molecular Surface Area (M.S.A.): Molecular surface calculation with 1.4 Å probe radius. This value is reciprocal to a Van der Waals surface area. Solvent Accessible Surface Area (S.A.S.A.): The surface area of the respective ligands accessible by a water molecule is presented. Polar Surface Area (P.S.A.): Solvent accessible surface area in the ligands contributed only by oxygen and nitrogen atoms. (a) Azadiradione in complex with 2WGS. (b) Azadiradione in complex with 1F61.

**Figure 5:**
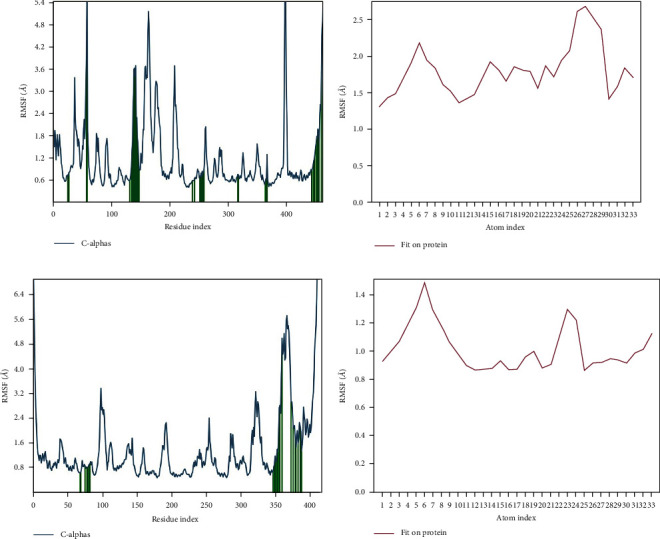
(a) R.M.S.F. plot of residues of the active site of 2WGS; (b) 1F61.

The R.M.S.D. plots of Azadiradione-2WGS and Azadiradione-1F61 complexes during the 100 ns of Molecular Dynamic Simulation have been shown in Figures 3(a) and 3(b). Figures 4(a) and 4(b) represent the R.M.S.F. plots, which have been used to study R.M.S.F. of the residues present in the active sites of proteins 2WGS and 1F61. R.M.S.F. plot (Figure 5(a) indicates the steepest value range at 50 and 400 residual indices, which implies increased fluctuation of the protein residues at these regions, signifying a high entropy value. While the R.M.S.F. plot (Figure 5(b) shows no steep value at 50 and 400 residual indices, a higher value was observed at ~350 residual indices.

Out of the different types of protein-ligand interactions observed, hydrogen bonds, hydrophobic interactions, and water bridges were the most prominent ones which played vital roles in stabilizing the interactions.

As part of the M.D. Simulation, R.M.S.D., Radius of Gyration (rGyr), Molecule Surface Area (M.S.A.), Solvent Accessible Surface Area (S.A.S.A.), and Polar Surface Area (P.S.A.) of ligands concerning the reference conformation were also examined and are presented in Figures 4(a) and 4(b). Root-Mean-Square Fluctuations (R.M.S.F.) were measured and plotted to quantify the flexibility of residues in the ligand-protein complexes. The R.M.S.F. of the protein-ligand complexes showed significant fluctuation for all the complexes. It deviated at many points in the 100 ns simulation period in both complexes, as shown in Figures 4(a) and 4(b).

Additionally, the hydrogen bonds between Azadiradione, a bioactive compound, and residues of the active site of the proteins were examined throughout the simulation. In the complex formed by 2WGS and Azadiradione, H-bonds were observed between the ligand, Azadiradione, and the residues SER-146, SER-151, TRY-153, and PHE-262 of 2WGS (Figure 5(a)). In the complex formed by 1F61 and Azadiradione, H-bonds were observed between Azadiradione and the residues, GLN-80, ARG-379, and ARG-386 of 1F61 (Figure 5(b)).

**Figure 6:**
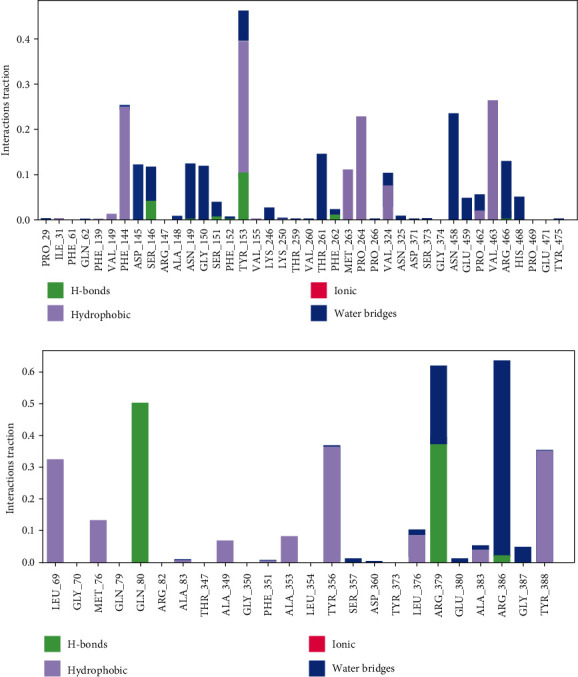
(a) Interaction diagram of 2WGS and Azadiradione during 100 ns M.D. Simulation. The green, blue, and purple colours represent H-bonding, water bridges, and hydrophobic interactions. (b) Interaction diagram of 1F61 and Azadiradione during 100 ns M.D. Simulation. The green, blue, and purple colours represent H-bonding, water bridges, and hydrophobic interactions.

From Figure 6(a), it was observed that among all the existing residues, Phe 144, Met 263, Pro 264, and Val 463, formed only hydrophobic bonds, and residues Tyr 153 and Val 324 predominantly formed hydrophobic bonds, along with other interactions. Residues Asp 145, Asn 149, Gly 150, Asn 458, Glu459, Arg 466, and His 468 formed only water bridges, and residues Ser 146, Ser 151, and Pro 462 predominantly formed water bridges, along with some other interactions. Residues Ser 146, Ser 151, Tyr 153, and Phe 262 formed H-bonds, along with some different types of interactions.  Figure 6(b) shows the formation of only a H-bond in Gln 80, and residues Arg 379 and Arg 386 formed H-bonds along with water bridges. Residues Leu 69, Met 76, Ala 349, and Ala 353 formed only hydrophobic bonds, and residue Gly 387 formed only water bridges. Predominant water bridges, along with H-bond, were formed in Arg 386.

### 2.4. ADME-Tox Study Using SWISS ADME

ADME-Tox study of Azadiradione exhibited noble drug-like properties [26–31]. ADME-Tox study results are presented in detail (Table 2).

The ADME-Tox analysis performed for the selected compound Azadiradione indicates that the compounds have an acceptable range of important physicochemical parameters such as Mw and Consensus Lipophilicity Score. In addition, it passed all the three major drug discovery rules: Lipinski, Ghose, and Veber rules. This ADME-Tox study revealed that the compound Azadiradione should not have any issue with becoming a successful drug candidate from the biochemical point of view. The computational docking study of the selected plant-based ligand against the target enzymes G.S. and I.C.L., followed by Molecular Dynamics Simulation studies and ADME-Tox study, is sufficient to predict the antitubercular nature of Azadiradione. Azadiradione is a triterpenoid compound used traditionally in several diseases and has some reports against malaria, respiratory disorders, cancer, intestinal helminthiasis, leprosy, constipation, inflammation, blood morbidity, dermatological complications, rheumatism, and so on [32, 33], which also supports its existing acceptability as a drug molecule.

## 3. Conclusion

In view of the wide-spectrum resistance of the currently existing M.D.R. and XDR T.B. strains display against the presently used antitubercular drugs, the present study was designed to dock 1778 compounds from four medicinal plants. *Azadirachta indica*, *Camellia sinensis*, *Adhatoda vasica,* and *Ginkgo biloba* have been selected against two targeted enzymes: Glutamine Synthetase and Isocitrate Lyase, of *M. tuberculosis* to identify new antitubercular agent(s). The *in silico* study revealed the binding efficacy of a compound from *Azadirachta indica,* namely, Azadiradione, towards the targeted enzymes, Glutamine Synthetase and Isocitrate Lyase, suggesting its potential inhibitory property towards the targets. M.D. Simulation study showed significant stability of the ligand-receptor complexes formed by Azadiradione (Table 3) with the two targeted enzymes.

Furthermore, the ADME-Tox study of Azadiradione exhibited noble drug-like properties of the ligand. The collective findings of the present *in silico* study, have been interpreted to propose that Azadiradione possesses significant potential as a candidate antitubercular molecule which is subject to future validation.

## 4. Materials and Methods

### 4.1. Hardware and Software Used

For the current study, a Lenovo computer unit with Linux and M.S. Windows operating systems, 1 T.B. S.S.D., 32 GB RAM supported by 8 GB graphics card was used. The software and tools used for different computational analyses were AutoDock Tools [34], AutoDock Vina version 1.1.2 [34–36], PyMol and Chimera Packages [37–40], Discovery Studio, and Schrödinger Suite version 2020-3. For Molecular Dynamics (M.D.) Simulation, the Desmond module provided by Schrödinger suite 2020-3 was used in a Linux platform supported by the above-mentioned hardware specifications. Other online resources used in the study have been mentioned in subsequent parts of the article.

### 4.2. Databases Used

Multiple databases were used at different stages of the study; one such significant database is the K.E.G.G. pathway, which includes the metabolic and regulatory pathways sections. Utilizing data from K.E.G.G. pathway database, the present study compared host metabolism with critical metabolic pathways of *M. tuberculosis*, and the necessary enzymes were identified [41]. Essential enzymes of the pathogen, *M. tuberculosis*, were also identified with the help of the Database of Essential Genes (D.E.G.). D.E.G. has reported 614 such essential genes [42].

3D structures of targeted proteins, that is, Glutamine Synthetase (Chain A, PDB ID: 2WGS) and Isocitrate Lyase (Chain A, PDB ID: 1F61) were retrieved from RCSB PDB database [14, 43–47]. Other databases like Drug Bank, PDB Bind, ZINC, and so on were also used in executing different steps of the research study, mainly to get the 2D and 3D information of the selected ligands.

### 4.3. Ligand Dataset Preparation

The active compounds from four different plants, *viz*., *Azadirachta indica, Camellia sinensis, Adhatoda vasica,* and *Ginkgo biloba* were collated to prepare a Ligand Library for the analysis. Databases like Drug Bank Database (https://go.drugbank.gov), ChEMBL, and PubChem (https://pubchem.ncbi.nlm.nih.gov) were used to obtain the structures of the small molecules for the preparation of the combinatorial library. A large number of previously reported phytochemicals in the four plants were also included in the library. 2D-structure trial and calculation of various chemical properties of the phytochemicals used in the present study were conducted in B.I.O.V.I.A. Draw of Dassault Systèms. T.O.R.S.D.O.F. utility in AutoDock Tools was used to set the ligands' default roots, rotatable bonds, and torsions.

### 4.4. Protein Structure Preparation

The three-dimensional structures of Glutamine Synthetase (PDB ID: 2WGS) and Isocitrate Lyase (RCSB PDB Identifier: 1F61) of *M. tuberculosis* were retrieved from R.C.S.B. Protein Data Bank and changed into P.D.B.Q.T. format. The protein structures were prepared by adding polar hydrogen bonds and removing hetero atoms, water molecules, and bound ligand(s) using AutoDock Tools utility. Only Chain A from 2WGS and 1F61 was selected for studying the docking interactions.

### 4.5. Molecular Docking Analysis

Protein-Ligand Docking study was carried out to understand the interaction of the two molecules with the ligands and determine the ligand(s) that would form complexes possessing minimal energy with the receptor. In docking analysis, a flexible receptor molecule has to be achieved. So by using the GRID module of Auto Dock v4.0, the flexible receptor molecule was achieved by setting a little residue. In contrast, AutoDock Vina with PyRx platform was used for calculating the affinity of small molecules against the targeted proteins Glutamine Synthetase and Isocitrate Lyase [48] (https://pyrx.sourceforge.io). The best protein-ligand complexes were then selected based on the energy conformation of the complexes. PyMOL software in PYTHON platform was used to analyse complex interactions, which illustrated hydrophobic interactions, ionic interactions, and hydrogen bonding between the receptors and the respective ligands.

### 4.6. Molecular Dynamics (M.D.) Simulation

The Desmond software module of Schrödinger suite 2020-3 was used for carrying out the M.D. Simulation. Simulations of 100 ns were performed to analyze the in silico stability of the complexes formed by 2WGS and 1F61 with G.S. and I.C.L. OPLS3 force field was used to study the 2WGS and 1F61 complexes in the explicit solvent system during M.D. Simulation. The atomic framework was solvated with crystallographic water (TIP3P) particles under orthorhombic intermittent limit conditions for 10 Å buffer region. In addition to the deletion of the overlapping water molecules, the entire structure of 34,255 atoms were neutralized by adding Na^+^ as counter ions. An ensemble of a Nose-Hoover thermostat and barostat was utilized to maintain a constant temperature of 300 K and a constant pressure of 1 bar (N.P.T.) [26]. Hybrid energy minimization algorithms were used, consisting of 1000 steps of steepest descent followed by conjugate gradient algorithms. M.M.G.B.S.A. analysis was done for both the complexes to calculate ligand binding energies using the Prime module of Schrodinger Suite 2020-3.

### 4.7. *In Silico* ADME-Tox Study

ADMET-Tox plays a vital role in the field of drug testing and design. Servers and software are available that use data from previously reported drugs to predict the ADMET properties of ligand molecules. *In silico* ADME-Tox studies were performed using a Swiss ADME server to identify the best-performing ligands [27–30]. Various Lipinski parameters were used to evaluate ligand(s)' drug-likeness and ADMET properties.

## Figures and Tables

**Table 1:** Docking results of Azadiradione with two enzymes GS and ICL.

Sl. No	Compound Name	Binding affinity of the ligands with glutamine synthetase				Binding affinity of the ligands with Isocitrate Lyase			
		Binding affinity (kcal/mol)	RMSD/UB	RMSD/LB	Number of H bonds formed (manually observed)	Binding affinity (kcal/mol)	RMSD/UB	RMSD/LB	Number of H-bonds observed
1	Azadiradione	-10.2	0	0	2	-8.5	0	0	2
2	7-Desacetyl-7-benzoylazadiradione	-10.1	0	0	2	-9	0	0	3
3	7-Desacetyl-7-benzoylgedunin	-9.1	0	0	2	-9.8	0	0	3
4	alpha-Amyrin	-8.8	10.503	6.395	2	-8	7.656	2.823	1
5	Gedunin	-8.7	0	0	2	-8.2	0	0	3

**Table 2:** Table of ADME-Tox study.

Compound	Oral bioavailability							Pharmacokinetic properties	LogKp (skin permeation)	Water solubility	Lipinski/Ghose/Veber (pass(Y)/fail (N))
	MW	cLogP	HBA	HBD	RB	TPSA (Å^2^)	B-score				
Azadiaradione	450.57 g/mol	4.3	5	0	3	73.58	0.55	GI absorption high	-5.60 cm/s	Insoluble	Y/Y/Y
7-Desacetyl-7-benzoylazadiradione	512.64 g/mol	5.38	5	0	4	73.58	0.17	GI absorption low	-4.83 cm/s	Poorly soluble	N/N/Y
7-Desacetyl-7-benzoylgedunin	544.63 g/mol	4.86	7	0	4	95.34	0.55	GI absorption high	-5.45 cm/s	Poorly soluble	Y/N/Y
alpha-amyrin	426.72 g/mol	7.05	1	1	0	20.23	0.55	GI absorption low	-2.51 cm/s	Poorly soluble	Y/N/Y
Gedunin	482.57 g/mol	3.73	7	0	3	95.34	0.55	GI absorption High	-6.25 cm/s	Moderately soluble	Y/N/Y

MW: molecular weight; cLogP: consensus lipophilicity score; HBA: H-bond acceptor; HBD: H-bond donor; RB: no. of rotatable bonds; PSA: polar surface area; B-score: bioavailability score; Lipinski/Ghose/Veber: rules of drug discovery.

**Table 3:** 2D structure and properties of Azadiaradione.

2D structure of Azadiaradione	Properties as calculated by BIOVIA Draw
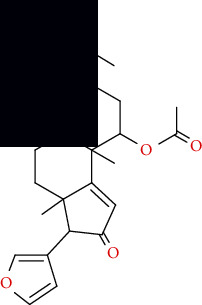	PSA: 73.58
	ALogP: 4.0905
	Stereo center count: 7
	Hydrogen acceptor count: 4
	Hydrogen donor count: 0
	Composition: C: 74.6% H: 7.6% O: 17.8%
	Formula weight: 450.57
	Exact mass: 450.24
	Molecular formula: C_28_H_34_O_5_

## Data Availability

The supporting data to the results reported in the article can be found with the corresponding authors, Dr. Saurov Mahanta and Dr. Saravanan Muthupandian.
